# Assessment of Knowledge and Practices of Standard Precaution against Blood Borne Pathogens among Doctors and Nurses at Adult Emergency Room in Addis Ababa, Ethiopia

**DOI:** 10.1155/2019/2926415

**Published:** 2019-04-23

**Authors:** Yohanis Asmr, Lemlem Beza, Hywot Engida, Tariku Bekelcho, Netsanet Tsegaye, Yibeltal Aschale

**Affiliations:** ^1^Department of Emergency Medicine and Critical Care Nursing, College of Medicine Health Science, University of Gondar, Gondar, Ethiopia; ^2^Department of Emergency Medicine and Critical Care Nursing, College of Medicine and Health Science, Addis Ababa University, Ethiopia; ^3^Department of Emergency Medicine and Critical Care Nursing, College of Medicine and Health Science, Arba Minch University, Ethiopia; ^4^Department of Medical Parasitology, College of Health Sciences, Debre Markos University, Debre Markos, Ethiopia

## Abstract

**Background:**

Standard precautions are infection control techniques against pathogenic microorganisms that are present in human blood and can cause disease in humans.

**Objective:**

This study aims to assess knowledge and practice of standard precautions against blood borne pathogens among doctors and nurses in adult emergency room, Addis Ababa, Ethiopia.

**Methods:**

Institutional based cross sectional study was conducted from February to March 2018. A total of 128 study participants selected from four public hospitals were enrolled in this study. Data were collected using standardized pretested questionnaire and thencoded, entered, checked for completeness, and analyzed using SPSS version-23 statisticalsoftware. Chi-square test was used to measure the association between variables.* P values* <0.05 were taken as statistically significant.

**Result:**

The mean knowledge score of standard precaution measures was 10.3 out of 14 knowledge items. Out of 32 doctors, 93.8% (n=30) have good knowledge and out of 91 nurses, 86.8% (n=79) have good knowledge. The mean practice level of the study subjects was 8.5 out of 12 practice items. Majority (73.6%) of nurses have good practice level than doctors (21.8%). Knowledge level was significantly associated with the presence of infection control officer, infection control guideline, and washing hands before touching patients. Profession, training, and the presence of infection control guideline in emergency room were significantly associated with practice level of respondents (P<0.05).

**Conclusion:**

Both nurses and doctors have good knowledge of standard precaution measures. However, nurses have better practice level than doctors. Orientation during employment and continuous training programs should be provided for the newly employed health workers. In addition sustainable supply systems should be available in each hospital management.

## 1. Background

Standard precautions are a set of precautionary measures designed to prevent transmissions of blood born infectious diseases [1]. Blood borne pathogens such as HIV, HBV, and HCV are the most serious and contributed to be a major threat in the workplace [2]. In practical terms, standard precautions include the use of gloves, aprons, goggles, suitable care of contaminated instruments (needles and sharps), house keeping with appropriate cleaning policies and ensuring strict adherence to standard practices. This needs provision of protective materials, proper training of health care providers, and adherence to sterilization and disinfection protocols [1, 3].

Occupational exposure to blood borne pathogens from needle sticks and other sharps injuries is a serious problem but it is often preventable [4]. The world health organization (WHO) estimated that, of the 35 million health care workers worldwide, three million experiences percutaneous exposures to blood borne pathogens each year. Among these exposed health care professionals, two million were exposed to HBV, 0.9 million to HCV, and 170,000 to HIV [2]. The incidence of needle stick injury in Ethiopia is 17.5% annually which is attributed to risky habit and inappropriate standard precaution compliance [5].

Standard precaution against blood borne pathogens refers to infection control techniques to pathogenic microorganism that are present in human blood and can cause disease in humans [3, 6]. Health care workers standard precaution against blood borne pathogens is essential components of any strategy to prevent infectious diseases [7]. Health care providers who are prone to infections with blood borne pathogens are nurses, laboratory technicians, surgeons, housekeepers, morgue technicians, and nonnursing attendants [8, 9]. Nurses are more likely to be exposed to microorganisms during their daily practice due to their frequent close contact with patients [10]. Doctors are also exposed to blood borne pathogens during blood work, physical examination and might develop infection [11].

The differences in knowledge of standard precaution by health care workers might be influenced by their variable type of training [12, 13]. Absence of an enabling environment in the health institution such as lack of constant running water or shortage of personal protective equipment can lead to poor practices with standard precaution [14, 15]. Compliance with standard precaution practice requires appropriate attitude of health workers over long periods of time demanding motivation and technical knowledge of them [1, 4]. In health institutions of developing countries like Ethiopia, hand washing practice even though not strict is among the components of infection prevention techniques [6, 15].

Standard precautions have been widely promoted in high-income countries to protect health care workers from occupational exposure to blood borne pathogens and the consequent risk of infection. Standard precautions are often practiced partially there by exposing the health care workers to unnecessary risk of infection with blood borne pathogens [2]. Even if Ethiopian Federal Minister of Health have clearly defined policies and procedures to implement standard precautions practice, less attention is given in facility level for the preventive strategies in reducing occupational injuries and infection. Assessing knowledge and practice of standard precautions against blood borne pathogens particularly in those health professionals working in emergency department is a life-saving procedure. There is no study conducted specifically on knowledge and practices of standard precaution against blood borne pathogens.

Therefore, this study is preliminarily intended to assess knowledge and practice of standard precautions against blood borne pathogens among doctors and nurses in the emergency room which is very important in differentiating the gap and implementing standard precautions against infectious pathogens. Findings from this study will help in planning and implementing appropriate interventions to improve compliance to standard precautions mainly related to blood borne pathogens among doctors and nurses.

## 2. Materials and Methods

### 2.1. Study Area

The study was conducted in Addis Ababa, Ethiopia, in four selected hospitals, Tikure Anbessa Specialized Hospital (TASH), Yekatit 12 Hospital Medical College (Y12 HMC), Menelik II Referral Hospital (Menelik II RH), and Zewditu Memorial Referral Hospital (Zewditu MRH) in adult emergency room. The TASH is one of the emergency centers in Addis Ababa which was established in 1973 during the regime of Emperor Haile Selassie. There is high patient flow where people get emergency medical and nursing care services. Zewditu MRH is also found in Central Addis Ababa. It is the leading hospital in the treatment of ART patients which currently treats over 6,000 patients each month and also gives general emergency health services. Menelik II RH and Yekatit 12 HMC are also the oldest hospitals which are providing general emergency management services and other health related services.

### 2.2. Study Design and Period

Institutional based cross sectional quantitative study was conducted in four hospitals found in Addis Ababa to assess knowledge and practice of standard precautions against blood borne pathogens among doctors and nurses from February 2018 to March 2018.

### 2.3. Study Population Characteristics

The source population of this study was all physicians and nurses who are working in each adult emergency department of four selected hospitals. All physicians (seniors, residents, and general practitioners) and nurses (Diploma, BSc, and MSc) who are working in adult emergency department and willing to participate in the study were enrolled in this study. Physicians and nurses who were not present in their work place during the study period were excluded from the study.

### 2.4. Sampling Techniques

The study was conducted in Addis Ababa in the four public hospitals. These hospitals were selected purposively. To obtain study subjects from selected hospitals, stratified random sampling method was used. Stratification was into two strata (doctors and nurses). Then, the number of doctors and nurses who work in adult emergency management room were proportionally allocated in accordance with the total number of source population obtained from each emergency department. Finally, the respondents were selected by simple random sampling technique using lottery method.

### 2.5. Sample Size Determination

The sample size was determined using single population proportion formula: [n=*z2p *(1−*p*)/d2] considering 95% CI and 50% prevalence which is 384. Since the total population is less than 10,000, the final corrected sample size was 128 using the population correction formula.

### 2.6. Data Collection Tools and Techniques

Data were collected using self-administrated structured questionnaire which was adopted from previous study conducted in North Wollo Zone in 2006 on assessment of knowledge attitude and practice of health care workers on standard precautions. The questionnaire was developed in English version and translated to Amharic version then back to English to check consistency of questions. The questionnaire consists of three parts: sociodemographic information, knowledge assessment, and practices assessment. Knowledge was assessed using 14 questions which include multiple choice and yes or no questions. Practices were assessed in a similar way using 17 questions.

### 2.7. Data Entry and Analysis

Data were coded, entered, checked for completeness and analyzed using SPSS version-23 statistical software. Respondents who scored greater than or equal to the mean score of knowledge questions were taken as good knowledge and responds who had greater than or equal to the mean score of practical questions were taken as good practices. Respondents who scored knowledge and practical questions below the mean were taken as poor knowledge and poor practices respectively. Descriptive statics were carried out to illustrate means, standard deviations, and frequency of the study variables. Chi-square test was used to measure the association between dependent and independent variables.

### 2.8. Data Quality Management

Data quality was managed during collection, coding, entry, and analysis steps. Pretest was conducted among 10% of the study population in Betel teaching hospital before the actual data collection to assess the reliability of data collection instruments and to avoid confusing questions. Then, corrective measures were taken on the data collection tools based on the pretest result. The data collectors were first oriented on standardized data collection, particularly in the proper filling of questionnaire. One senior supervisor who monitored each level of data collection was assigned in each selected hospital to ensure weather the data collectors follow the proper and preplanned method of data collection or not. Data were then collected by the trained data collectors under close supervision of the supervisors to improve the quality. The supervisor and principal investigator has checked each collected data daily to make sure that whether all questions are properly filled or not.

## 3. Result 

### 3.1. Sociodemographic Characteristics

From a total of 128 doctors and nurses, 123 fulfilled the inclusion criteria and participated in the study with the response rate of 96.1%. Among these respondents, 66 (53.7%) were males and 64 (52%) were in the age range from 22 to 27 years (mean age 28 years). The majority (74.0%) have less than 5 years of work experience (Table 1).

### 3.2. Training Status of the Study Participants

Among the study participants, 77 (62.6%) have taken training on infection prevention. Of which, 67 (87.1%) were nurses and 10 (12.9%) were doctors. Less than half (37.4%) of the respondents have not taken training on infection prevention at all.

### 3.3. Knowledge on Availability of Infection Prevention Officer and Guideline

About 64.2% (n=79) of the respondents are aware of the presence of infection prevention officer and 35.8% (n=44) are not aware. About 62% (n=76) of the respondents are aware of the presence of infection prevention guideline, whereas 38% (n=47) are not aware of any guideline.

### 3.4. Knowledge on Standard Precaution against Blood Borne Pathogens

About 98.4% (n=121) of study participants reported that needle was one type of waste discarded in safety box. Majority (85.4%) of study participants reported that they wash their hand with soap and water. Majority (51.2%) of the study participants have reported that they wash their hands always after touching the patient. About 36.6% (n=45) of study participants reported a history of splashing and 60.2% (n=74) have no history of splashing (Table 2).

### 3.5. Mean Knowledge Score of the Study Participants

**Figure 1:**
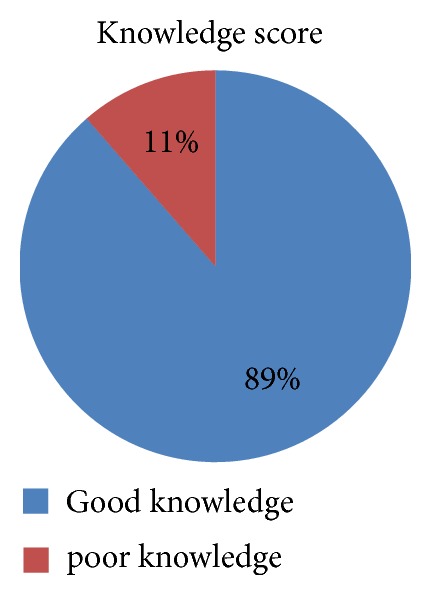
Knowledge score of study participants in selected hospitals.

The mean knowledge score of all participants was 10.3 out of 14 knowledge items. About 89% of study participants have good knowledge (Figure 1).

### 3.6. Practice Level of Participants on Standard Precaution against Blood Borne Pathogens

Majority (28.5%) of study participants do not wash their hands due to inaccessibility of hand washing materials. About 65% (n=80) of study participants have used personal protective equipment before touching the patients. The majority (95.1%) of the study participants apply “use and throw” method after using of materials like nasal cannula, prong, and face mask. About 79 (64.2%) have reported that they have decontaminated laryngoscope after using it for intubation (Table 3).

### 3.7. Practice Score of Study Participants

**Figure 2:**
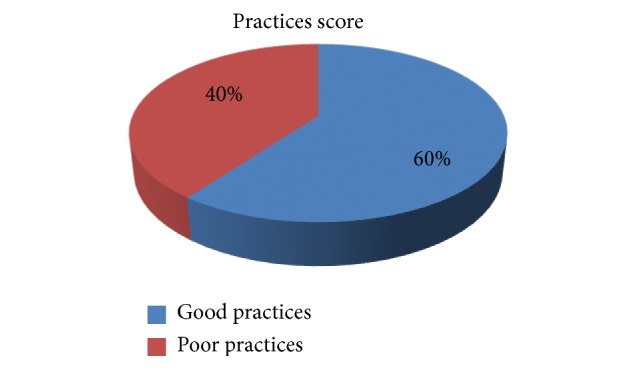
Practices score of the study participants in selected hospitals.

The mean practice score of study participants was 8.56 out of 12 practices items. About 60% of study participants have good practice (Figure 2).

### 3.8. Factors Associated with Knowledge and Practice Level

There was significant associations between knowledge score of study participants and presence of infection control officers, infection prevention guidelines, and hand washing before touching patients (P<0.05) (Table 4).

Statistically significant association was observed between practice score and professional categories, training, presence of infection control guideline, wearing personal protective equipment before touching patients and washing hands before touching the patients (P<0.05) (Table 5).

## 4. Discussion

The finding of this study showed that the mean knowledge score of all participants was 10.3 out of 14 knowledge items and the mean score of practice was 8.56 out of 12 practice items. The overall knowledge score for both categories were 88.6% and the remaining (11.4%) of study participants had poor knowledge score. About 94.5% of doctors had good knowledge whereas 87% of nurses had good knowledge score. This is comparable with a study conducted in South East Nigeria in which about 97.0% of doctors had good knowledge score and 92.0% nurses had good knowledge score [8]. This similarity might be due to the fact that standard precautions have been incorporated in the nursing and medical student curriculum.

The overall practice score for both categories was 60.2% good practices and 39.8% poor practices. In this finding, nurses had good standard precautions practice against blood borne pathogens which accounted for 74% as compared to doctors (21.8%). This result is slightly higher than a study conducted in Southeast Nigeria among nurses and doctors, which has 75% good practices for nurses and only 15% for doctors [8]. This difference might be due to the difference in sample size and sampling methods.

In the recent study conducted in India of tertiary care hospitals on the knowledge, attitude and practice of standard precautions among medical and nursing students, nursing students had better knowledge compared with medical students which was 85.6% in nursing students and 75.6% in medical students. This might be responsible for the generally better compliance to standard precautions practice observed among nurses compared to other health practitioners [16].

In the present study 62.6% of the study participants had training on infection prevention which is directly related to infection prevention. Of which, 87% were nurses and the remaining 13% were doctors. This is slightly higher than a study conducted in TASH which is 49.2% [6] and in North Wollo Zone, Amhara region, by which only 45.8% of the respondents had prior training [17]. This difference might be due to lack of national guideline and learning materials on infection prevention in local language for health care workers, clients and communities and also could be due to absence of continuous support and supervision to improve the standard precaution.

According to this study there was no significant knowledge difference between male (89.0% had good knowledge score) and female (88.0% had good knowledge score) on standard precautions against blood borne pathogens. This is different from a study conducted in Jamaica which was highest among women compared with men, and among nurses (90.0%), compared with medical doctors (88.0%) [12].

In this study only 43.1% of the respondents have used personal protective equipment after sustaining needle stick or sharp injuries which is higher than the study conducted in TASH among emergency medicine professionals in which 24.6% of respondents used personal protective equipment after sustaining needle stick or sharp injuries [6]. This might be due to the difference in sample size and sampling methods.

According to this study 98.4% of the respondents have worn gloves during invasive procedures. However, a study conducted in Ghana, Accra Hospital, showed that 88% of respondents indicated that they have worn gloves routinely when performing invasive procedures on patients. This study demonstrated better practice in use of gloves in emergency rooms than the practice of Ghana Accra hospital health professionals [3]. This could be fear of the infectiousness of diseases like HIV and HBV due to their higher prevalence in Ethiopia.

Even though gloves, gowns, aprons, masks, and goggles are advised by the world health organization to help protect health care workers and clients from blood borne infections including HIV, in this study only 88.8% respondents worn utility gloves, 76.8% worn mask for invasive procedures, and 36.3% put on goggles. But study done in perceptions and practice of standard blood and body fluid precautions by registered nurses at a major Sydney teaching hospital showed that 84% of the respondents wear gloves face masks for invasive procedures. The least practiced is wearing of protective eye shields (24%)[18]. This showed significant difference because of inaccessibility and absence of personal protective device particularly goggles and faces masks in emergency room.

In this study 36.6% of study participants have experienced blood born body fluid splash to mucus membrane. This is comparable with a study conducted in SNNPR in which 32.4% experienced blood born body fluid splash on the mucus membrane [19]. The results of the present findings were obtained in a hospital with a high patient flow and intensive health care services, whereas the previous study included health care workers working in health centers, which provide less intensive health care than hospitals [2].

Based on this study 61.5% of nurses have washed their hands always after touching patient intact skin. This finding is different from a study conducted in Thailand in which 75% of doctors and 47% of nurses have washed their hands after caring for patients, and 16% of doctors and 50% of nurses rubbed their hands with alcohol after washing them with an antiseptic [20]. Low level of hand washing practice among doctors in this study might be attributed to the absence of information during introductory on job training courses and orientation. In this study significantly higher number of trained study participants had good compliance of hand washing practices than those who had no training on infection prevention (P<0.05).

## 5. Conclusion and Recommendation

The study has demonstrated good knowledge of standard precaution against blood borne pathogens in both doctors and nurses. Nurses exhibited a significantly higher compliance to standard precaution practices compared to doctors. Training staffs on standard precautions, principles, and practice needs to be implemented to provide the necessary knowledge on compliance to standard precaution practices. In addition, strict supervision, operational guideline and on-job training courses and orientation need to be implemented regularly.

## Figures and Tables

**Table 1:** Sociodemographic characteristics of doctors and nurses in selected hospitals, February 19 to March 31;, 2018.

Variables		Doctors (n=32)		Nurse (n=91)		P-value
		Number	Percent	Number	Percent	
Age group (year)	22-27	13	40.6	51	56	0.15
	28-33	16	50	30	33	
	34-39	2	6.3	5	5.5	
	>40	1	3.1	5	5.5	
	Total	32	100	91	100	

Sex	Male	20	62.5	46	50.5	0.04
	Female	12	37.5	45	49.5	
	Total	32	100	91	100	

Marital status	Divorced	1	3.1	1	1.1	0.09
	Married	11	34.4	40	44	
	Single	20	62.5	50	54.9	
	Total	32	100	91	100	

Work experience	<5	28	87.5	63	69.2	0.34
	5-10	4	12.5	23	25.3	
	>10	0	0	5	5.5	
	Total	32	100	91	100	

**Table 2:** Knowledge of study participants on standard precautions against blood borne pathogens in selected hospitals, February to March 2018.

Variables	Frequency	Percent
*Handwashing after touching patient intact skin*		
Always	63	51.2
Often	15	12.2
Sometimes	39	31.7
Never	6	4.9

*Reason for reuse of syringe and needle*		
Shortage of supply	23	18.7
Knowledge deficit	67	54.5
Carelessness	24	19.5
To reduce cost of treatment	9	7.3

*Had blood or body fluid splash to eye, mouth, or nose*		
Yes	45	36.6
No	74	60.2
Do not remember	4	3.3

*Measures taken after exposure to blood and body fluids*		
Wash with soap and water	105	85.4
Wash with alcohol, iodine, chlorine	42	34.1
Visiting VCT	80	65.0
Seek PEP	74	60.2
Report to head person	61	49.6
Others *∗*	25	20.3

*Source of infection *		
Health personnel	87	70.7
Contaminated medical equipment	111	90.2
Contaminated air	93	75.6
Other patients	62	50.4
Others*∗∗*	24	19.5

N.B: Others*∗*=consulting physicians; others*∗∗*=insect and small animals.

**Table 3:** Practice level of study participants on standard precaution against blood borne pathogens among study participants in selected hospitals, February to March 2018.

Variables	Frequency	Percent
*Reasons for not washing hands*		
Inaccessibility of hand washing materials	35	28.5
Not always necessary	24	19.5
Absence of hand washing materials	18	14.6
Emergency condition	30	24.4
I use glove	16	13

*Wearing PPE before touching the patient*		
Yes	80	65
No	43	35

*Device used as PPE*		
Apron	37	30.1
Mask	59	47.9
Utility glove	71	57.7
Gown	61	49.6
Eye protector glove	29	23.6
Boots shoes	42	31.1

*How many times you sustained needle stick injury*		
1 times/year	27	69.2
2 times/year	9	23.1
>3 times/year	3	7.7

*Reuse of medical equipment's*		
Yes	6	4.9
No	117	95.1

*Giving or decontaminate laryngoscope after use*		
Yes	79	64.2
No	44	35.8

**Table 4:** Factors associated with knowledge level of the study subject in selected hospitals from February to March 2018.

Variables	Response	Knowledge level				P value
		Poor		Good		
		N	%	N	%	
Training	No	8	17.4	38	82.6	0.105
	Yes	6	7.8	71	92.2	

Profession	Doctor	2	6.2	30	93.8	0.862
	Nurse	12	13.2	79	86.8	

Infection control officer	No	9	20.5	35	79.5	0.018*∗*
	Yes	5	6.3	74	93.7	

Infection control guideline in emergency room	No	9	19.1	38	80.9	0.033*∗*
	Yes	5	6.6	71	93.4	

Wearing gloves during invasive procedure	No	0	0	2	100	0.609
	Yes	14	16.6	107	88.4	

Discarded, used material as per standard precaution guideline	No	2	5.3	36	94.7	0.153
	Yes	12	14.1	73	85.9	

Reused needle or syringe	No	8	9	81	91	0.176
	Yes	6	17.6	28	82.4	

Wash hands before touching the patients	No	11	17.2	53	82.8	0.035*∗*
	Yes	3	5.1	56	94.9	

Wearing personal protective equipment's before touching the patients	No	7	16.3	36	83.7	0.21
	Yes	7	8.8	73	91.3	

Have you ever had NSI	No	8	9.5	76	90.5	0.34
	Yes	6	15.4	33	84.6	

**Table 5:** Factors associated with practice level of the study participants in selected hospitals from February to March 2018.

Variables	Response	Practice level				P value
		Poor		Good		
		N	%	N	%	
Training	No	36	78.3	10	21.7	<0.05*∗*
	Yes	26	33.8	44	55.7	

Profession	Doctor	25	78.1	7	21.9	<0.05*∗*
	Nurse	24	26.4	67	73.6	

Infection control officer	No	27	61.4	17	38.6	0.07
	Yes	35	44.3	44	55.7	

Infection control guideline in emergency room	No	32	68.1	15	31.9	0.002*∗*
	Yes	30	39.5	46	60.5	

Wearing gloves during invasive procedure	No	1	50	1	50	0.991
	Yes	61	50.4	60	49.6	

Discarded used material as per standard precaution guideline	No	27	71.1	11	28.9	0.002*∗*
	Yes	35	41.2	50	58.8	

Reused needle or syringe	No	35	39.3	54	60.7	<0.05*∗*
	Yes	27	79.4	7	20.6	

Wash hands before touching the patients	No	42	65.6	22	34.4	<0.05*∗*
	Yes	20	33.9	39	66.1	

Wearing personal protective equipment's before touching the patients	No	36	83.7	7	16.3	<0.05*∗*
	Yes	26	32.5	54	67.5	

Have you ever had NSI	No	36	42.9	48	57.1	<0.05*∗*
	Yes	26	66.7	13	33.3	

## Data Availability

The data used to support the findings of this study are available from the corresponding author upon reasonable request.

## Abbreviations

HIV: Human Immunodeficiency Virus
HBV: Hepatitis B Virus
HCV: Hepatitis C Virus.
